# Multidimensional monitoring of anaerobic/aerobic azo dye based wastewater treatments by hyphenated UPLC-ICP-MS/ESI-Q-TOF-MS techniques

**DOI:** 10.1007/s11356-016-7075-5

**Published:** 2016-06-21

**Authors:** Benjamin Frindt, Jürgen Mattusch, Thorsten Reemtsma, Axel G. Griesbeck, Astrid Rehorek

**Affiliations:** 1 Faculty of Applied Natural Sciences, University of Applied Sciences, Cologne, TH Köln, Kaiser-Wilhelm Allee, 51368 Leverkusen, Germany; 2 Helmholtz-Centre for Environmental Research, Department of Analytical Chemistry, Permoser Str. 15, 04318 Leipzig, Germany; 3 Department of Organic Chemistry, University of Cologne, Greinstr. 4, 50939 Köln, Germany

**Keywords:** ICP-MS/ESI-Q-TOF coupling, Online toxicity measurements, Biological azo dye treatment, Predicted reaction mechanisms and toxicity, Transformation products, Decolorization, DOC removal

## Abstract

Sulfonated reactive azo dyes, such as Reactive Orange 107, are extensively used in textile industries. Conventional wastewater treatment systems are incapable of degrading and decolorizing reactive azo dyes completely from effluents, because of their stability and resistance to aerobic biodegradation. However, reactive azo dyes are degradable under anaerobic conditions by releasing toxic aromatic amines. To clarify reaction mechanisms and the present toxicity, the hydrolyzed Reactive Orange 107 was treated in anaerobic-aerobic two-step batch experiments. Sulfonated transformation products were identified employing coupled ICP-MS and Q-TOF-MS measurements. Suspected screening lists were generated using the EAWAG-BBD. The toxicity of the reactor content was determined utilizing online measurements of the inhibition of *Vibrio fischeri*. The OCHEM web platform for environmental modeling was instrumental in the estimations of the environmental impact of generated transformation products.

## Introduction

The remaining dyes from several industrial sources (e.g., textile, dye and dye intermediates, recycling, pharmaceuticals, etc.) are regarded as dischargers of a variety of organic pollutants into natural water resources or wastewater treatment systems (Carmen and Daniela 2012). Due to the high quantities of water used in the dying process, the textile industry is one of the biggest producers of liquid effluent pollutants (Saratale et al. 2011). Estimates show that 280,000 tons of textile dyes are discharged in industrial effluents per year worldwide (Jin et al. 2007). One group of these dyes are the sulfonated reactive azo dyes, which contain chromophoric azo groups, where nitrogen atoms are linked to sp^2^-hybridized carbon atoms of aromatic rings with additional sulfonic acid groups (Pathak et al. 2014). Plenty of treatment methods are applied to reduce the high amounts of colored wastewater. Chemical treatments (oxidation, electrolysis, ozonation) and physical treatments (filtration, adsorption, coagulation/flocculation) are generally applied, though biological treatments have the main advantage of converting over 70 % of organic matter (expressed by COD) to biosolids (Anjaneyulu et al. 2005). However, reactive textile dyes are not degradable in conventional aerobic treatment plants as they are persistent to biological oxidative degradation (Easton 1995). Yet, a degradation and decolorization of reactive azo dyes can be achieved under anaerobic conditions due to the cleavage of the azo bonds, with a further release of potential toxic and carcinogenic aromatic amines (Sweeney et al. 1994). With respect to the biological transformation products, several analytical LC-MS methods were reviewed for the analysis of sulfonated compounds (Reemtsma 2003) to clarify degradation/transformation mechanisms. Therefore, the coupling of ICP-MS and Q-TOF-MS for element and structure-specific identifications could be one further step in analyzing sulfonated transformation products. For monitoring the environmental impact of biologically treated substances, online toxicity measurements could be useful to improve long-term treatments in bioreactor systems. In addition, postulations of reaction mechanisms and interpretations of a present toxicity could be improved using several modeling databases.

## Material and methods

### Reactive azo dye: C.I. Reactive Orange 107

The industrial textile dye C.I. Reactive Orange 107 (CAS 90597–79-8) was kindly provided by DyStar (Leverkusen, Germany) for biological experiments. The hydrolyzed form of Reactive Orange 107 was prepared for 4 h at 80 °C after adjustment to pH 11 with 1 M L^−1^ sodium hydroxide and was neutralized with 1 M L^−1^ hydrochloride acid before treating.

### Treatment system

The biological treatment was realized with an anaerobic-aerobic CSTR bioreactor system with a volume of 30 L each, according to previous studies of Plum and Rehorek (2005). Anaerobic and aerobic recirculating sludges were obtained from the industrial wastewater treatment plant of Currenta GmbH & Co. OHG (Leverkusen, Germany). The biomass was characterized by a dry matter of 25 g L^−1^ (DIN EN 12880 2001a), a sludge volume index of 20.6 mL g^−1^ (DIN EN 14702 2006) and by determining the loss on ignition of dry mass of 81.6 % (DIN EN 12879 2001b). The biomass was immobilized on polyurethane foamed carriers coated with activated carbon provided by LEVAPOR GmbH (Leverkusen, Germany) to improve biotransformation efficiency in both reactors, as reported in several studies (Yoo 2000; Pearce 2003; Srinivasan and Viraraghavan 2010; Vijayaraghavan et al. 2013; Kumar and Mongolla 2015). The bacterial consortium was conditioned for 10 days until immobilization rates >90 % were measured by determining the decreasing dry matter of the sludge according to DIN EN 12880 2001a. The pH was automatically adjusted to 7.0 ± 0.2 in both reactors. The temperature of the anaerobic treatment step was maintained at 38 ± 0.5 °C. Peristaltic pumps continuously recirculated the reactor content (100 L h^−1^) through ultrafiltration membrane cells in cross flow mode. The polyethylene membrane had a molecular weight cutoff of 50,000 Da, and the utilization and recovery rates for the observed compounds were tested in previous experiments by Plum and Rehorek (2005) and Rehorek et al. (2006). In addition to the online analytical set-up from Rehorek et al. (2006), an online UV-Vis spectrometer probe was installed with a flow cell in by-pass of the anaerobic bioreactor system for continuous decolorization measurements. Due to the involved cleaning procedure of the spectro::lyser probe (scan Messtechnik GmbH, Vienna, Austria), in combination with bacterial immobilization leading to a decreasing turbidity, spectrometric measurements could be carried out during the complete treatment time. The decolorization was calculated with the evaluation method (arithmetic mean from the spectral absorption coefficient of 436, 525, and 620 nm) of Döpkens and Krull (2004) according to the German wastewater regulation appendix 38. Furthermore, the actual DOC (dissolved organic carbon) could be measured online with the QuickTOC from LAR Process Analysers AG (Berlin, Germany).

In this study, 2 mM L^−1^ of the hydrolyzed Reactive Orange 107 was treated for 10 days under anaerobic conditions with a subsequent aerobic treatment of 7 days. The redox potential (against hydrogen potential) was continuously monitored during the anaerobic batch experiments. For Reactive Orange 107, a threshold of −330 mV for an anaerobic decolorization could be determined.

### UPLC-ICP-MS/ESI-Q-TOF-MS system

The determination of the transformation products of the hydrolyzed Reactive Orange 107 was performed employing the coupled UPLC-ICP-MS/ESI-Q-TOF-MS system.

No special pretreatment except filtration was applied to the analyzed samples. The retention times of the coupled ICP-MS and Q-TOF systems were adjusted by analytical standards of dyes without biological matrix. The biologically treated samples were filtered with a pore size <0.2 μm. Therefore, nylon syringe filters were used in offline experiments, and recovery rates for the observed compounds were determined. For a fast chromatographic separation of the substances of interest, an UPCL Series Infinity 1290 (Degasser, binary pump, thermostated autosampler) was used. This system was coupled with an ICP-QQQ-MS (Agilent 8800) for element specific analysis and a Q-TOF-MS (Agilent 6530) for accurate mass determination in parallel by splitting the mobile phase 1:1 with the help of a flow splitter (Analytical Scientific Instruments, Inc., California, USA). The mobile phase was prepared with ULC/MS grade methanol (methanol absolute Bisolve BV, Valkenswaard, The Netherlands) and ammonium acetate (for HPLC <99.0 %, Fluka). A poroshell solid phase with a porous microparticulate column packing was used to achieve high-resolution results as solid phase. The injection volume was set to 20 μl. For the detection of the sulfonated compounds after the flow splitter, the quadrupole Q1 was set to *m*/*z* 32 for pre-selection the ion of interest (S^+^) followed by a reaction with O_2_ in the collision/reaction cell to form SO^+^. The quadrupole Q3 was fixed at *m*/*z* 48 to measure the product ion. The conditions for the separation and measurements are listed in Table 1.

**Table 1:** Analytical set-up and measurement conditions for UPLC-ICP-MS/ESI-Q-TOF-MS analysis

Conditions		
LC	Agilent 1290 Infinity	
Column	Agilent Poroshell 120 EC-C18 3 × 150 mm 2.7 μm	
Flow rate	0.7 mL min^−1^	
Mobile phase	Eluent A	1 mM NH_4_Ac in H_2_O
	Eluent B	5 mM NH_4_Ac in MeOH
Gradient	0–8 min	99.5 % A
	8–25 min	99.5–60 % A
	25–35 min	60 % A
	35.1–40 min	99.5 % A
ESI*-*Q*-*TOF*-*MS	Agilent Q-TOF 6530	
Ionization	Negative mode	
Fragmentor voltage	175 V	
Capillary voltage	3000 V	
Mass range	100–600 amu	
Gas temperature	350 °C	
Drying gas	11 L min^−1^	
Nebulizer pressure	30 psi	
Sheath gas temperature	400 °C	
Sheath gas flow	10 L min^−1^	
Collision energy	0 eV	
ICP*-*MS	Agilent 8800 ICP-QQQ	
RF power	1550 W	
Option gas	10 % (80 % Ar/20 % O_2_)	
Cell gas (O_2_)	0.35 mL min^−1^	
Sample depth	7 mm	
Elemental analysis	Detection: Q1	m/z 32 (S^+^)
	Detection: Q3	m/z 48 (SO^+^) product ion
Integration time	1 s	

### Databases used for the prediction of biotransformation and environmental impact of biological azo dye wastewater treatments

The EAWAG-BBD (Biocatalysis/Biodegradation Database: Swiss Federal Institute of Aquatic Science and Technology, Duebendorf, CH; University of Minnesota) was used to create suspected lists for the spectroscopic screening of anaerobic and aerobic transformation products. The database contains information on microbial biocatalyzed and biodegradation pathways for primarily xenobiotic environmental pollutants (Ellis and Wackett 2012). With the primary focus on metabolic reactions, additional information on enzymes can be obtained via links to the Kyoto (Kanehisa et al. 2002), ExPASy (Gasteiger et al. 2003) and BRENDA (Scheer et al. 2011) databases. The pathway predictions are carried out by biotransformation rules, based on known chemical reactions of functional groups, like reduction, oxidation, elimination, or hydrolysis (Wicker et al. 2010).

The OCHEM web platform (online chemical modeling environment) was used to interpret the toxicity measurements during the biological treatment (Sushko et al. 2011). Besides biological activity and physicochemical properties of compounds, the database can predict information on toxicology and ecotoxicology effects for the bacteria *Tetrahymena pyriformis*, the most ciliated model used for laboratory research (Sauvant et al. 1999). In environmental toxicity prediction studies against *T. pyriformis*, a wide collection of 1093 experimental measurements were used for a critical assessment of QSAR (quantitative structure-activity relationship) models (Tetko et al. 2008).

### Online toxicity measurements

During the textile dyeing process, hydrolysis (reaction that occurs in the presence of water) takes place in a competitive reaction to the dye fixation (Christie 2015). This leads to the hydrolyzed dye (RO107_Hydrolyzate_), that does not have any affinity with the fibers to form covalent bonds (Christie 2015), resulting in high concentrations in textile wastewater. Gottlieb et al. (2003) could show increasing toxicity for hydrolyzed dyes compared to the parent form with the bioluminescent bacterium *Vibrio fischeri* in a previous study. The decolorization of azo dyes under reductive conditions leads to aromatic amines, which can accumulate in the anaerobic treatment step (Brown and Laboureur 1983; Plum and Rehorek 2005; Phugare et al. 2011). In several studies, the increasing toxicity due to the release of aromatic compounds could be proven in separate offline toxicity measurements (Wang et al. 2002; Gottlieb et al. 2003; Işik and Sponza 2004; Sponza 2006; Işik and Sponza 2007; Solís et al. 2012). Therefore, the challenge was to develop an online toxicity measurement system, which can monitor the actual toxicity in the bioreactor during the complete treatment. The actual toxicity inside the reactor systems was measured with the Microtox® CTM (continuous toxicity monitor) from Modern Water (Cambridge, UK) with the bioluminescent bacterium *V. fischeri*. The common use of the instrument, according to the manufacturer, is the monitoring of rivers, lakes, reservoirs, seawater, recycled water, and groundwater (Modern Water, 2012). Toxic effects were determined by the inhibition of the bioluminescence of *V. fischeri*, measured with detectors inside the instrument after a contact time of 2 min. Offline samples of dyes and their transformation products were analyzed with the CTM and compared to common respiration tests with activated sludge. Although the results from respiration tests are more significant to evaluate real toxicity towards activated sludge bacterial community, evidenced correlations of measured and compared inhibitory effects provide the use of this online monitoring system. In addition, Gutiérrez et al. (2002) could show a higher sensitivity for bioluminescence inhibition in a comparative study between an offline Microtox® and respiration tests for seven organic toxic compounds.

**Fig. 1:**
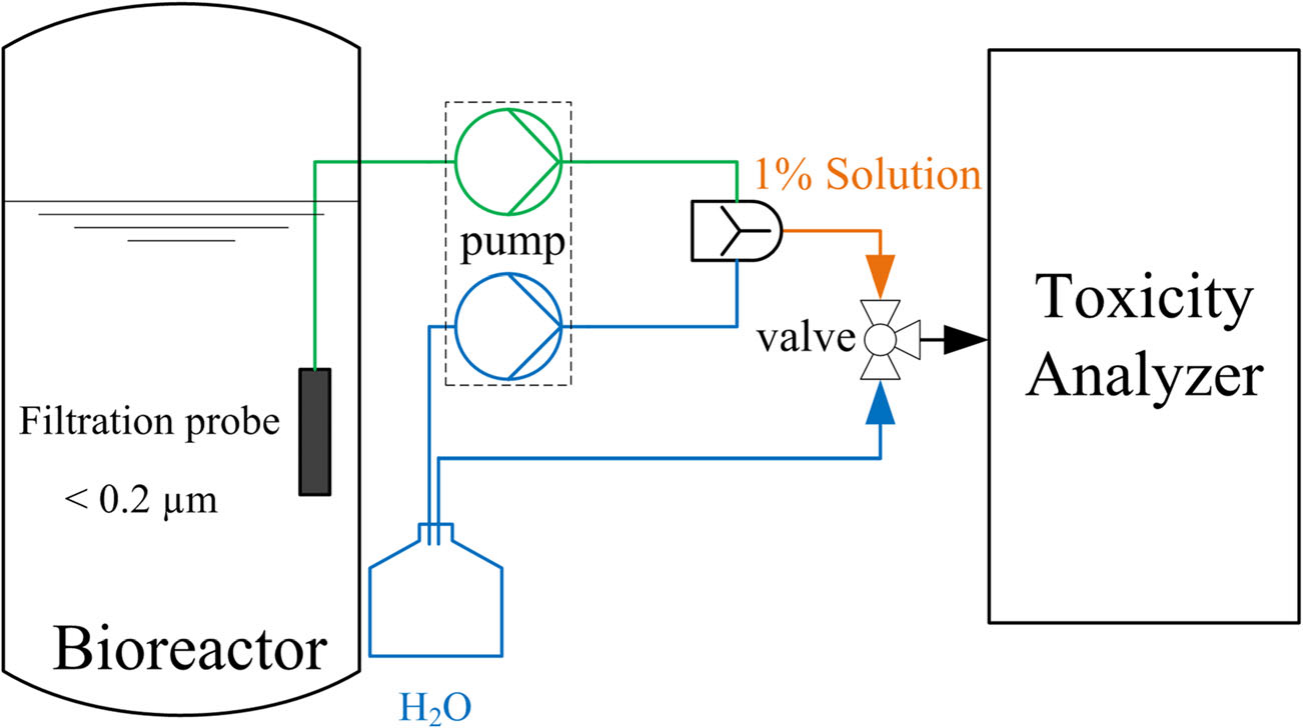
Online toxicity measurement set-up

Due to the high sensitivity of the *V. fischeri* within the toxicity analyzer, the samples from the bioreactor systems had to be filtrated and diluted. The final sample preparation and dilution system is shown in Fig. 1. The in situ sampling was performed with a filtration probe from Trace Analytics GmbH (Braunschweig, Germany), to receive sterile samples free of solids (<0.2 μm). A multichannel peristaltic pump from Ismatec (Wertheim-Mondfeld, Germany) was utilized to pump the sample and dilute it 1:100 with distilled water to a 1 % solution inside a static mixer with a coupled debubbler. The dilution was carried out using different inner diameters of the peristaltic pump tubes to obtain a total sample flow of 3.5 mL min^−1^.

The current inhibition was calculated based on DIN EN ISO 11348-3 for freeze-dried bacteria, shown in Eq. 1, where the light output prior to the exposure to the sample (*L*_0_) and the light output after the exposure (*L*_t_) at time *t* are given.

1 $$ \mathrm{Inhibition}\ \left[\%\right]=\frac{L_0-{L}_t}{L_0}\times 100 $$

To compensate the decreasing light output during the whole treatment/measurement process according to Michaelis-Menten kinetics for *V. fischeri*, reference values with distilled water had to be created for online measurements. Therefore, an electropneumatic valve was integrated to receive reference values (*L*_0_) while measuring. The cycle time for sample (*L*_*t*_) and distilled water (*L*_0_) was set to a ratio of 45 and 15 min measuring time. Fifteen minutes was determined as a minimum switching time to receive constant inhibition values tested with 3.5-dichlorophenol from Sigma-Aldrich Chemie GmbH (Munich, Germany). An internally developed software was used to calculate the actual inhibition in real-time to monitor the toxicity during the treatment process. With this measurement set-up, online toxicity could be continuously measured for 4 weeks according to the decreasing bioluminescence of *V. fischeri*.

## Results and discussion

### Compound analysis by UPLC-ICP-MS/ESI-Q-TOF-MS techniques

**Fig. 2:**
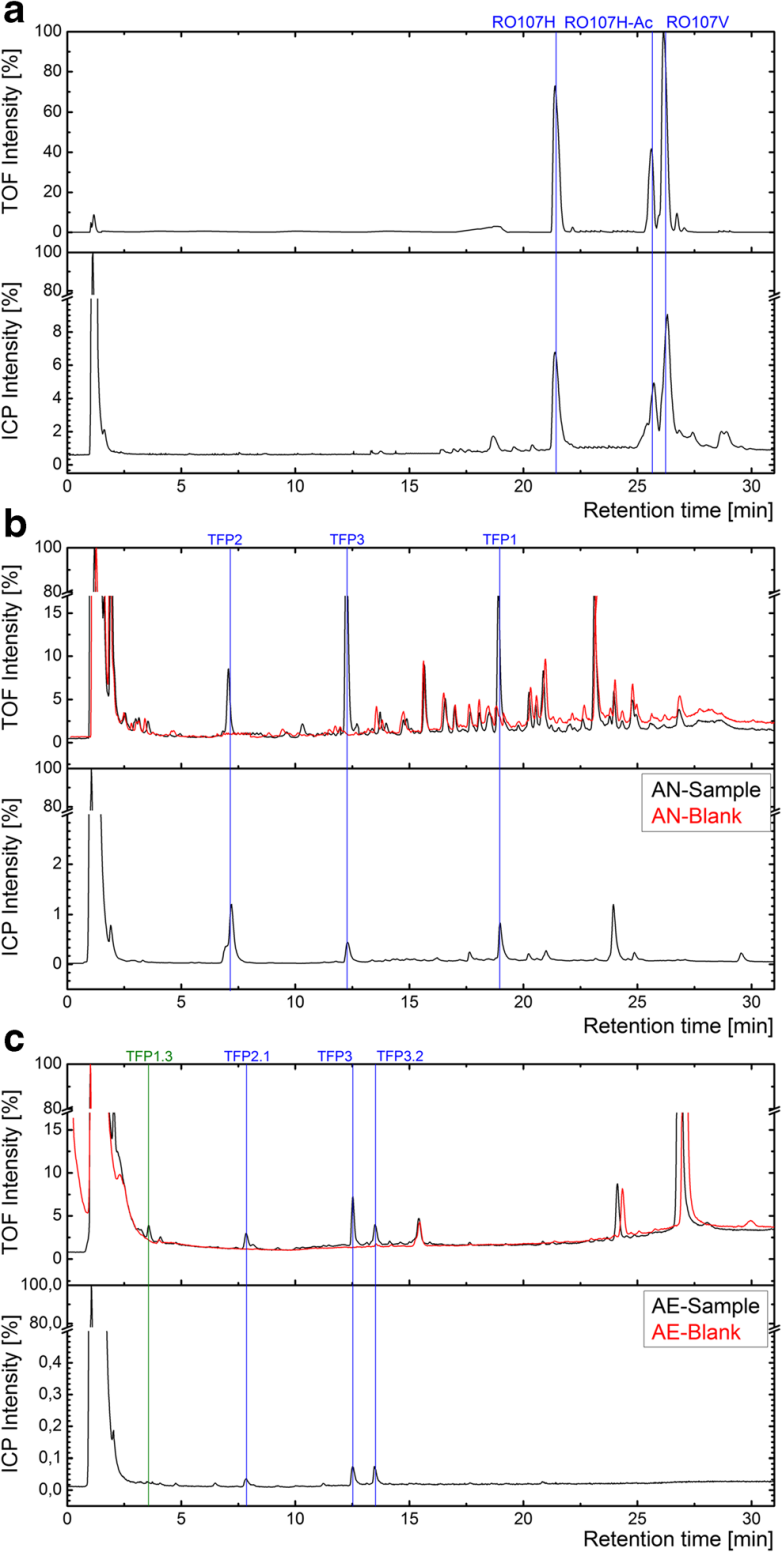
UPLC-ICP-MS/ESI-Q-TOF-MS chromatograms for the parent compounds after the hydrolysis of Reactive Orange 107 (**a**), anaerobic-treated transformation products (**b**), aerobic-treated transformation products (**c**) in matrix in relative intensities. The *blank* (matrix) is shown in *red* chromatograms and the treated samples in *black* chromatograms

Figure 2 shows the UPLC-ICP-MS/ESI-Q-TOF-MS chromatograms of the analyzed samples. The observed exact masses and mass shifts with their ion formulas and abbreviations are listed in Table 2. Pane A indicates the parent compounds after the hydrolysis of Reactive Orange 107. Three masses of interest could be found after the hydrolysis of the initial dye with retention times of 22.9 to 27.2 min. All sulfonated dye forms could be indicated by their ICP-MS (m/z 48 SO^+^) peaks in the chromatogram. The compounds were determined as [M-H]^−^ product ions in negative ionization mode with a maximum mass shift of −4.07 ppm. The second pane B shows the chromatograms of the anaerobic treated dye forms (black) in matrix (red). Because of the absence of spectrometric peaks in the specific retention times of the parent compounds, it is reasonable to assume that complete reductions of all azo bonds occurred under anaerobic conditions. Furthermore, each signal and compound peak in the TOF-analysis could be assigned to sulfonic substances, because of their significant ICP-MS peaks. With respect to the generated suspected screening list from EAWAG-BBD pathway prediction system, three anaerobic transformation products could be found regarding to their exact measured masses. With an absolute mass shift <5 ppm in combination with the known parent molecules, the transformation products could be determined as [M-H]^−^ product ions (Russell and Edmondson 1997; Storm 2002). In the subsequent aerobic treatment (Fig. 2, pane C), the measured matrix samples indicated less sulfonic compounds compared to the anaerobic matrix. After blank subtraction (of the matrix), four peaks were found in the TOF-signal of the sample after the aerobic treatment. The observed masses with their actual retention times could indicate one aerobic accumulating transformation product (TFP3) in this analysis. In addition, three further biological degraded compounds were classified as suspected compounds, suggested by the EAWAG-BBD pathway prediction system. A missing ICP-MS peak for TFP1.3 indicated a desulfonation of one anaerobic generated aromatic compound. The product ions of this sample could be determined as [M-H]^−^ ions, where TFP3.2 was found as a [2M-H]^−^ ion within an acceptable mass shift.

**Table 2:** Exact masses and mass shifts of the found compounds of the biologically treated samples with ion formula and abbreviations

Compounds of the hydrolyzed dye					
Abbr.	Ion formula	m/z_exp_	m/z_calc_	Δm/z [ppm]	Sulfur identification
RO107H	C_16_H_18_N_4_O_7_S_2_	442.0617	442.0635	−4.07	Yes
RO107H-Ac	C_14_H_16_N_4_O_6_S_2_	400.0511	400.0525	−3.50	Yes
RO107V	C_16_H_16_N_4_O_6_S_2_	424.0511	424.0526	−3.54	Yes
Transformation products after anaerobic treatment					
TFP1	C_8_H_11_N_3_O_4_S	245.0470	245.0482	−4.89	Yes
TFP2	C_8_H_11_NO_3_S	201.0460	201.0468	−3.98	Yes
TFP3	C_6_H_9_N_3_O_3_S	203.0365	203.0373	−3.94	Yes
Transformation products after subsequent aerobic treatment					
TFP1.3	C_8_H_9_NO_4_	183.0532	183.0536	−2.19	No
TFP2.1	C_8_H_10_O_5_S	218.0249	218.0259	−4.59	Yes
TFP3	C_6_H_9_N_3_O_3_S	203.0365	203.0373	−3.94	Yes
TFP3.2	C_6_H_6_O_6_S	205.9885	205.9894	−4.37	Yes

### Degradation/transformation mechanisms of hydrolyzed Reactive Orange 107

Reactive Orange 107, as an aromatic compound with one –N=N– group, constitutes one representative of the largest class of synthetic dyes in commercial applications (Zollinger 2003). As an anionic monoazo reactive dye, Reactive Orange 107 has a sulfonate group (–SO_3_H) as an auxochrome group, which is ionizable and confers the binding capacity onto the textile material for the dye (Welham 2000).

**Fig. 3:**
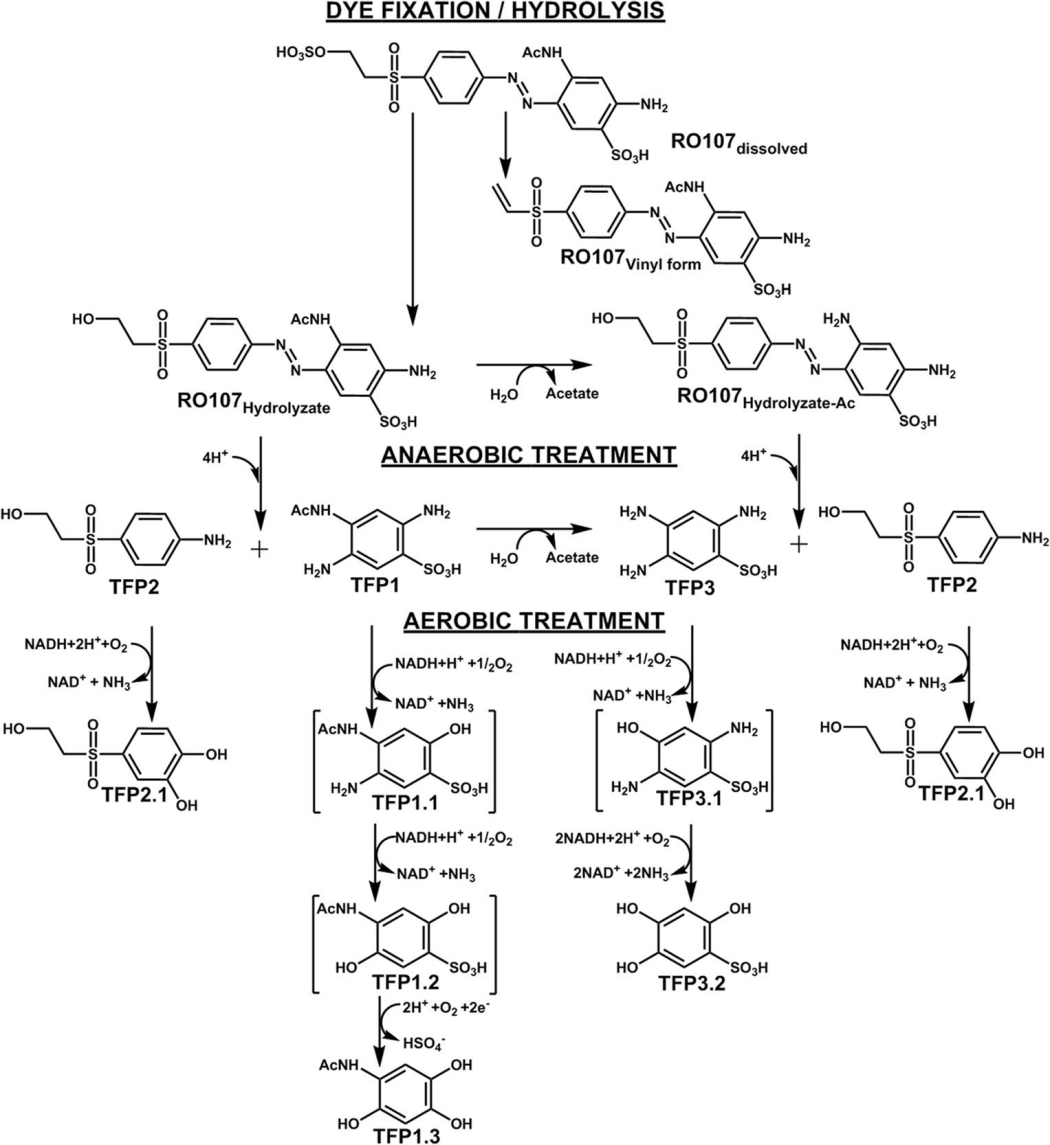
Proposed hydrolyzation/degradation mechanisms of Reactive Orange 107 under anaerobic and aerobic conditions

According to the dye fixation on textile fibers, the uptake of Reactive Orange 107 in fibers depends on a covalent bond with an additional electrostatic interaction with different charges of dye ion and fiber (chemisorption) (Carmen and Daniela 2012). In alkaline conditions, at high temperatures Reactive Orange 107 forms a reactive vinyl sulfone (–SO_3_–CH=CH_2_) group (RO107_Vinyl form_), that creates a bond with the fiber (Rehorek et al. 2006). In addition to the dye fixation with a nucleophilic substitution, hydrolysis takes place in a competitive reaction (Christie 2015). This leads to the hydrolyzed dye (RO107_Hydrolyzate_), which does not have any affinity with the fibers to form covalent bonds (Christie 2015). After the final washing process, hydrolyzed dye is discharged in the wastewater in high amounts compared to unfixed dye material (dos Santos et al. 2004). For this reason, the degradation mechanisms are mainly postulated and shown for the hydrolyzed Reactive Orange 107. Within the dye fixation/hydrolysis, an additional dye form could be found (RO107_Hydrolyzate-Ac_) (Rehorek et al. 2006). Due to hydrolysis, the acyl group is substituted, which could lead to an acetic elimination (Fig. 3).

In the anaerobic treatment step the cleavage of the azo bond could be observed for RO107_Hydrolyzate_ and RO107_Hydrolyzate-Ac_ through a decolorized sample. Under reductive conditions, 2-[(4-Aminophenyl)sulfonyl]ethanol (TFP2) was determined as a transformation product of both present dye forms. In addition, 2,4,5-triaminobenzenesulfonic acid (TFP3) was identified as a common transformation product of both dye forms. For the hydrolyzed RO107, 4-acetamido-2,5-diaminobenzenesulfonic acid (TFP1) could be determined as a primary breakdown product, which leads to TFP3 by hydrolysis of the acyl group. The eliminated group should be present as acetate due to the pH of the bioreactor system. All postulated transformation products could be observed with the LC-MS system (Fig. 2b).

In the subsequent aerobic treatment step, the anaerobic accumulated TFP2 was metabolized to the catechol 4-((2-hydroxyethyl)sulfonyl)benzene-1,2-diol (TFP2.1) by dioxygenase with loss of ammonia. Under aerobic conditions, the amine could be substituted completely, as described in previous studies (Viliesid and Lilly 1992; Blümel et al. 1998; Deniz and Grady 2003; Wang et al. 2006), so that TFP2 could not be found at the end of the aerobic treatment step (Fig. 2c).

For TFP1, a further elimination of the acyl group was observed under aerobic conditions. By acting on carbon-nitrogen bonds, amidohydrolase could lead to 2,4,5-triaminobenzenesulfonic acid (TFP3) under aerobic conditions (Hart and Orr 1975). In two subsequent steps, the transformation of TFP3 could be postulated. In several stages, the auxochrome amino groups was substituted by monooxygenase (Balba et al. 1979; Storm 2002; Stüber 2005), which leads to the aerobic accumulating 2,4,5-trihydroxybenzenesulfonic acid (TFP3.2), that was determined after the aerobic treatment.

In a parallel occurring transformation of TFP1 due to dioxygenase and monooxygenase, TFP1.1 and TFP1.2, which could not be determined, could be generated under loss of ammonia from the amine groups. Therefore, N-(2,4,5-trihydroxyphenyl)acetamide (TFP1.3) was found as an aerobic accumulating transformation product. The desulfonation of the aromatic sulfonate (TFP1.2) leading to the corresponding phenol (TFP1.3) could occur under sulfate limiting conditions, where aromatic sulfonates can be used as a primary s-source (Luther and Soeder 1987; Zürrer et al. 1987; Wittich et al. 1988; Kertesz et al. 1994; Ruff et al. 1999; Storm 2002). Compared to previous studies (Gottlieb et al. 2003; Supaka et al. 2004; dos Santos et al. 2007; Saratale et al. 2011; Balapure et al. 2014), a complete subsequent ring fission could not be observed in this experiment, although the treatment time of 7 days was much higher compared to conventional industrial wastewater treatment plants or lab-scale experiments (Işik and Sponza 2004; Spagni et al. 2010; Jonstrup et al. 2011; Muda et al. 2011).

### Results of online toxicity measurements by *V. fischeri* during the treatment process

**Fig. 4:**
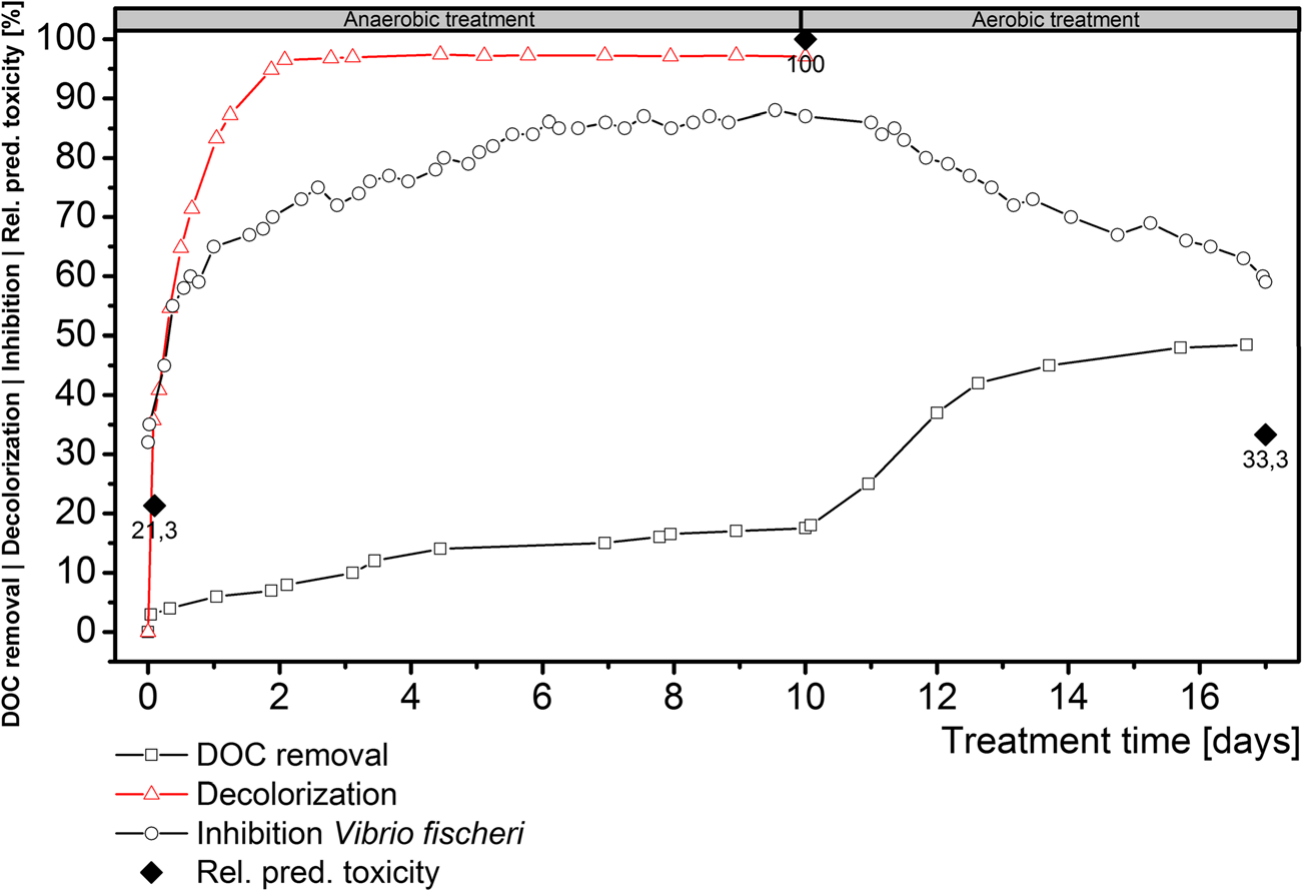
DOC removal, decolorization, *Vibrio fischeri* inhibition, and relative predicted toxicity of the biological treatment of RO107 hydrolyzed wastewater

With respect to the environmental impact of this biological azo dye treatment, the actual inhibition of *V. fischeri* could be determined during the whole two-step treatment process. As shown in Fig. 4, the toxicity in the anaerobic reactor system was demonstrated to increase in line with the decolorization due to the reductive cleavage of the available azo bonds. With the release of aromatic amines in this treatment step, the inhibition is increasing from 32 to 87 % within 10 days. This effect could be explained by the higher hydrophobicity of the amines, enabling more efficient passage through the cell membranes, resulting in elevated toxicity (Erkurt et al. 2010). A decolorization of 95 % was achieved during the first 2 days. However, a further anaerobic treatment in a total of 10 days achieved a decolorization of 97 %. Furthermore, the toxicity was reduced in the aerobic treatment step from 87 to 59 % within a treatment time of 7 days, due to the fade of aromatic amines. No additional decolorization or recolorization was determined in the aerobic treatment step. Related to the measured DOC, the anaerobic treatment led to a DOC removal of 17.5 % in 10 days. The subsequent aerobic degradation could induce a DOC removal of 48.5 % in total. These measurements showed a partial mineralization in the aerobic milieu, which could indicate a removal of the aromatic amines in this treatment step, used as an applied detection method in previous studies (Wiesmann et al. 2002).

Next to the online measured sum parameters, the relative predicted toxicity values for the reactor content are shown in Fig. 4. The toxicity for *T. pyriformis* was calculated with the OCHEM web platform for each observed compound. The toxicity could be determined as a concentration of a compound that inhibits 50 % growth in aqueous medium (IGC_50_). For mathematical analysis, the logarithm of the inverse of a toxic concentration (log(IGC_50_^−1^)) is used, which leads to large log(IGC_50_^−1^) for toxic compounds (Tetko et al. 2008). In combination with the relative compound concentration from the UPLC experiments, a relative predicted toxicity could be calculated for the hydrolyzed RO107, the anaerobic transformation products, and the subsequently observed aerobic transformation products. Table 3 shows the specific toxicity values for each observed compound. The predicted toxicity values correlated with the inhibition values of *V. fischeri* obtained online during the whole treatment process.

**Table 3:** DOC, predicted toxicity, and relative concentration of the hydrolyzed dye forms and the biological transformation products

Compound	Pred. toxicity [log(IGC_50_^−1^)/mg L^−1^]	Relative concentration [%]	Rel. pred. toxicity [%]	DOC [mg L^−1^]
Hydrolyzed RO107				
RO107H	128.0 ± 1.50	36.7	21.3	3450
RO107H-ac	17.1 ± 1.40	15.8		
RO107V	52.3 ± 1.40	47.5		
Anaerobic transformation products				
TFP1	341.0 ± 1.30	20.8	100.0	2846
TFP2	575.0 ± 0.82	46.1		
TFP3	255.0 ± 1.30	33.1		
Aerobic transformation products				
TFP1.3	298.0 ± 0.90	10.5	33.3	1776
TFP2.1	48.7 ± 0.93	23.8		
TFP3	255.0 ± 1.30	42.6		
TFP3.2	15.6 ± 1.10	23.1		

## Conclusion

The coupled ICP-MS and ESI-Q-TOF-MS systems were used to advance screening methods for the identification of sulfonated transformation products. With the help of the Q-TOF-data, the proposed molecules from the EAWAG-BBD were confirmed employing exact mass measurements. Ninety-seven percent decolorization was achieved under anaerobic conditions cleaving the present azo bonds. With the release of aromatic amines, an increasing toxicity compared to the initial dye product was determined with online measurements. Subsequently, decreasing toxicity in the aerobic treatment step was detected and could be explained by the fade of aromatic amines. Predicted toxicity values for the biological transformation products using the OCHEM web platform correlated with the inhibition values obtained employing online measurements. These results indicate that biological treatment of dyestuff can only be effective with the combination of anaerobic and aerobic treatment steps mediating efficient decolorization and detoxification.
